# Seasonal Prevalence and Molecular Characterization of *Cryptosporidium* spp. in Dairy Cattle of Inner Mongolia, Northern China

**DOI:** 10.3390/life16081221

**Published:** 2026-07-23

**Authors:** Chun-Hao Zhang, Tao Liu, Jun-Tao Jia, Zhen Liu, Chun-Xue Zhao, Yi-Yang Liu, Yue-Rong Lv, Shi-Yuan Xue, Yu-Lin Ding, Li Zhao, Yong-Hong Liu

**Affiliations:** 1 College of Veterinary Medicine, Inner Mongolia Agricultural University, Hohhot 010018, China; 18522891734@163.com (C.-H.Z.); lt163db@163.com (T.L.); 13514761934@163.com (C.-X.Z.); 18702243475@163.com (Y.-Y.L.); lyr765619420@163.com (Y.-R.L.); xsy55145600@163.com (S.-Y.X.); dingyulin2001@126.com (Y.-L.D.); 2 Vocational and Technical College, Inner Mongolia Agricultural University, Baotou 014109, China; juntaojia1982@imau.edu.cn; 3 Agriculture and Animal Husbandry Bureau of Ordos, Ordos 017000, China; liuzhen0906@163.com

**Keywords:** *Cryptosporidium*, prevalence, seasonal variations, Inner Mongolia, cattle

## Abstract

**Background:** *Cryptosporidium* spp. are zoonotic pathogens that threaten animal husbandry and public health. Systematic data on the seasonal distribution of *Cryptosporidium* in dairy cattle of Inner Mongolia, China, are lacking. This study investigated the effects of seasonal variation on the prevalence and genetic diversity of *Cryptosporidium* in dairy cattle. During the four seasons of 2024, 505 fecal samples were collected from an intensive dairy farm in Hohhot, Inner Mongolia. *Cryptosporidium* species were identified using nested PCR targeting the SSU rRNA gene, and *C. parvum* subtypes were determined by *gp60* sequencing. **Results:** The overall prevalence was 39.21% (198/505), with significant seasonal variation (spring 36.57%, summer 16.22%, autumn 45.38%, winter 55.38%; χ^2^ = 41.37, *p* < 0.001). No significant differences were observed among age groups or between diarrheic (40.48%) and nondiarrheic (38.58%) cattle. Five species were identified: *C. andersoni* (46.46%), *C. bovis* (23.74%), *C. ryanae* (16.16%), *C. parvum* (12.12%), and *C. suis* (1.52%). Two *C. parvum* subtypes were detected: IIdA20G1 (79.17%) and IIdA19G1 (20.83%). **Conclusions:** This is the first systematic investigation of the seasonal distribution of *Cryptosporidium* in cattle in Inner Mongolia and the first detection of *C. suis* in Chinese cattle. Both *C. parvum* subtypes detected are zoonotic, highlighting the need for public health surveillance.

## 1. Introduction

*Cryptosporidium* spp. are common intestinal protozoan parasites that can infect various vertebrates, including humans [1]. These pathogens are primarily transmitted via the fecal–oral route, including direct contact with infected hosts or contaminated fomites, as well as indirectly through contaminated water, food, or environmental surfaces [2]. The clinical manifestations of cryptosporidiosis depends on the infecting *Cryptosporidium* species involved and host-related factors. Infected hosts may present with a spectrum of symptoms ranging from asymptomatic carriage to acute diarrhea, malnutrition, and weight loss. Disease severity of clinical disease is generally associated with host age and immune status, with young animals and immunocompromised individuals being more susceptible to severe or life-threatening infections [3].

To date, 48 valid *Cryptosporidium species* and ~120 genotypes have been identified worldwide [4]. Among these, 23 species and 2 genotypes have been reported in humans [5,6], whereas at least 12 species have been identified in cattle [7]. In China, at least 10 *Cryptosporidium* species have been reported in cattle, of which the four major species are *C. andersoni*, *C. bovis*, *C. ryanae*, and *C. parvum* [8]. Furthermore, *C. parvum*, a major zoonotic species, and *C. hominis*, which is generally considered as an anthroponotic species but has occasionally been detected in cattle, have also been reported, with *C. parvum* being the most predominant [9]. However, *C. hominis* is primarily adapted to humans, and there is currently no evidence that it causes clinical disease in cattle. In most countries, zoonotic cryptosporidiosis is primarily caused by *C. parvum* subtype family IIa; however, in China, bovine *C. parvum* infection is exclusively attributed to subtype family IId [10].

Molecular-based studies on *Cryptosporidium* spp. in cattle have revealed a global pooled prevalence of 26.5% [11]. In China, the pooled prevalence is estimated at 17.0% or 11.9% [7,12]. Worldwide prevalence of *C. parvum* infection in dairy calves has been reported to be 21.9% [13]. Numerous countries across the world have explored the effects of seasonal variations on *Cryptosporidium* prevalence, including Denmark [14], Thailand [15], New Zealand [16], and Egypt [17]. However, only two studies have systematically investigated seasonal variation in *Cryptosporidium* prevalence in China, and only one of these focused on dairy cattle [18,19]. Hence, the present study was conducted to systematically determine the effects of seasonal variation on the prevalence, species distribution, zoonotic risk, and occurrence of *Cryptosporidium* in dairy cattle of Inner Mongolia, northern China. Notably, this single-farm design limits the generalizability of the findings to the broader regional cattle population.

## 2. Materials and Methods

### 2.1. Study Areas and Sample Collection

Four fecal sampling periods were conducted in late March, June, September, and December 2024, according to the climatic characteristics and seasonal division of the Northern Hemisphere. In total, 505 fresh fecal samples were collected from an intensive farm located in Hohhot, Inner Mongolia, China (111.253540° E, 40.572130° N). All sampled animals were female Holstein dairy cattle. Based on the different ages of the cattle, they are divided into the following four age groups: preweaned calves (0–60 days, *n* = 85), postweaned calves (61–180 days, *n* = 137), young cattle (181–360 days, *n* = 137), and adult cattle (≥361 days, *n* = 146). Preweaned calves (0–60 days) were kept in individual hutches, whereas older cattle were maintained in group pens. The herd was a closed population with on-farm replacement heifers and year-round calving, with peak calving occurring during autumn and winter. No shared surface water sources or contact with adjacent pig farms were present. Each animal was sampled only once, and no repeated sampling of individual animals was performed across seasons. During each sampling period, 5–20% of the animals in each age group were randomly selected using Excel RAND applied to the electronic ear-tag inventory. Owing to the dynamic nature of the herd (routine calving, culling, and intergroup transfers), the exact total herd size and age-group population at each sampling time point were not documented, and the precise sampling fractions could not be retrospectively determined. Of the 505 samples, 168 (33.27%) were obtained from diarrheic cattle and 337 (66.73%) from nondiarrheic cattle. In terms of collection method, 308 (60.99%) samples were directly collected from the rectum using a sterile gloved hand and 197 (39.01%) from the inner surface of fresh feces on the ground immediately following defecation. For the 197 ground-collected samples, the corresponding individual cattle were identified via direct observation of defecation and immediately matched to their unique ear-tag numbers to ensure accurate traceability. Relevant information was recorded in detail during the sampling process, including the age of cattle and the presence or absence of diarrhea. Samples were stored at 4 °C before being transported to the laboratory for DNA extraction.

### 2.2. DNA Extraction

Fecal DNA extraction was performed on the 505 samples with the E.Z.N.A^®^ Stool DNA Kit (Omega Biotek, Norcross, GA, USA) inside a biological safety cabinet in strict accordance with the supplier’s guidelines; the isolated material was then kept at −20 °C for downstream applications.

### 2.3. Polymerase Chain Reaction Amplification

*Cryptosporidium* spp. were identified by nested PCR of the SSU rRNA gene [20]. For the primary PCR, primers 18SiCF2 (5′-GACATA TCATTCAAGTTTCTGACC-3′) and 18SiCR2 (5′-CTGAAGGAGTAAGGAACAACC-3′) were used to amplify a 763-bp fragment. In the secondary PCR, 1-μL primary product and nested primers 18SiCF1 (5′-CCTATCAGCTTTAGACGGTAGG-3′) and 18SiCR1 (5′-TCTAAGAATTTCACCTCTGACTG-3′) were used to amplify a 587-bp fragment. SSU cycling: 95 °C for 5 min; 35 cycles of 94 °C 30 s, 57 °C 30 s, 72 °C 40 s; and 72 °C for 10 min (primary), or 30 cycles with 55 °C annealing (secondary). Each 25-µL reaction contained 12.5-µL 2× Taq Plus Master Mix II (Dye Plus), 0.5 µL of each primer (20 µM), 1-µL DNA template, and 10.5-µL distilled water.

For *C. parvum* subtyping, nested gp60 PCR was conducted using the primary primers LX0374 (5′-TTACTCTCCGTTATAGTCTCC-3′) and LX0375 (5′-GGAAGGAACGATGTATCTGA-3′), followed by the secondary primers AL3532 (5′-TCCGCTGTATTCTCAGCC-3′) and AL3534 (5′-GCAGAGGAACCAGCATC-3′), which amplified an 850-bp fragment [21]. Each 25-μL reaction mixture contained 12.5-μL Premix Taq™ (Ex Taq™ version 2.0 Plus Dye), 0.5 μL of each primer (20 μM), 1-μL of DNA template, and 10.5-μL distilled water. *gp60* cycling: 94 °C for 5 min; 35 cycles of 94 °C 45 s, 50.4 °C (primary) or 54 °C (secondary) 45 s, 72 °C 1 min; and 72 °C for 7 min. For samples with unsuccessful amplification, primers AL3532 (forward) and AL3533 (reverse, 5′-GAGATATATCTTGGTGCG-3′) were used [22], yielding a ~450-bp fragment. Positive and negative controls were included in each PCR run. The subtyping of *C. parvum* was accomplished by sequencing the PCR-positive products [23]. Products were sequenced bidirectionally at Sangon Biotech (Shanghai, China).

### 2.4. Sequence Analysis

Raw sequences were first edited and aligned against GenBank references (http://www.ncbi.nlm.nih.gov (accessed on 15 March 2026)) in MEGA 11.0 (http://www.megasoftware.net/ (accessed on 16 March 2026)). Species, community, and genotype identities were subsequently assigned via the Basic Local Alignment Search Tool (https://blast.ncbi.nlm.nih.gov/Blast.cgi (accessed on 17 March 2026)). To infer evolutionary relationships among the isolates, phylogenetic analysis was conducted on the SSU rRNA gene sequences. For *C. parvum* subtyping, the gp60 locus was analyzed. A maximum likelihood tree was reconstructed under the Tamura three-parameter model using MEGA 11.0, and the reliability of the branching patterns was assessed by bootstrap analysis with 1000 replicates.

### 2.5. Statistical Analysis

Statistical analyses were conducted in SPSS 26.0 (IBM Corp., Armonk, NY, USA). Univariable analyses (chi-square test and pairwise odds ratios) were used to examine the associations between *Cryptosporidium* infection and season, age group, and diarrhea status. Multivariable logistic regression was not performed due to the single-farm design and the cross-sectional sampling strategy. A two-tailed *p*-value below 0.05 was adopted as the threshold for statistical significance.

## 3. Results

### Cryptosporidium Infection Status, Species Distribution, and Subtypes

A total of 198 out of 505 dairy cattle tested positive for *Cryptosporidium* spp., yielding an overall infection rate of 39.21%. Seasonal infection rates varied considerably: spring was 36.57%, summer was 16.22%, autumn was 45.38%, and winter was 55.38%. The prevalence was significantly higher in spring than that in summer (odds ratio [OR] = 2.98, 95% confidence interval [CI]: 1.61–5.51, *p* = 0.0004) but lower than that in winter (OR = 0.46, 95% CI: 0.28–0.76, *p* = 0.002). The prevalence in summer was significantly lower than those in autumn (OR = 0.23, 95% CI: 0.13–0.43, *p* < 0.001) and winter (OR = 0.16, 95% CI: 0.08–0.29, *p* < 0.001). There were no statistically significant differences in prevalence between spring and autumn (OR = 0.69, 95% CI: 0.42–1.14, *p* = 0.145) or between autumn and winter (OR = 0.67, 95% CI: 0.41–1.09, *p* = 0.107).

Preweaned calves showed the highest positivity (45.88%, 39/85), followed by young cattle (43.07%, 59/137), postweaned calves (39.42%, 54/137), and adults (31.51%, 46/146), though these differences did not reach statistical significance (χ^2^ = 6.079, df = 3, *p* = 0.108). The infection risk in preweaned calves was higher than that in adult cattle, with a statistically significant difference (OR = 1.843, 95% CI: 1.062–3.199, *p* = 0.041); however, no statistically significant differences in infection risk were found between postweaned calves and adult cattle (OR = 1.414, *p* = 0.205) or between young and adult cattle (OR = 1.644, *p* = 0.059).

Diarrheic and nondiarrheic cattle exhibited comparable infection rates of 40.48% (68/168) and 38.58% (130/337), respectively, with no evidence of a statistically significant association (χ^2^ = 0.100, df = 1, *p* = 0.752) (Table 1).

All sampled animals were female Holstein dairy cattle from a single intensive dairy farm in Hohhot, Inner Mongolia, China. Five *Cryptosporidium* spp., viz., *C. andersoni*, *C. parvum*, *C. bovis*, *C. ryanae*, and *C. suis*, were identified by the sequence analysis of 198 PCR-positive products, with prevalence rates of 46.46% (92/198), 12.12% (24/198), 23.74% (47/198), 16.16% (32/198), and 1.52% (3/198), respectively.

**Figure 1:**
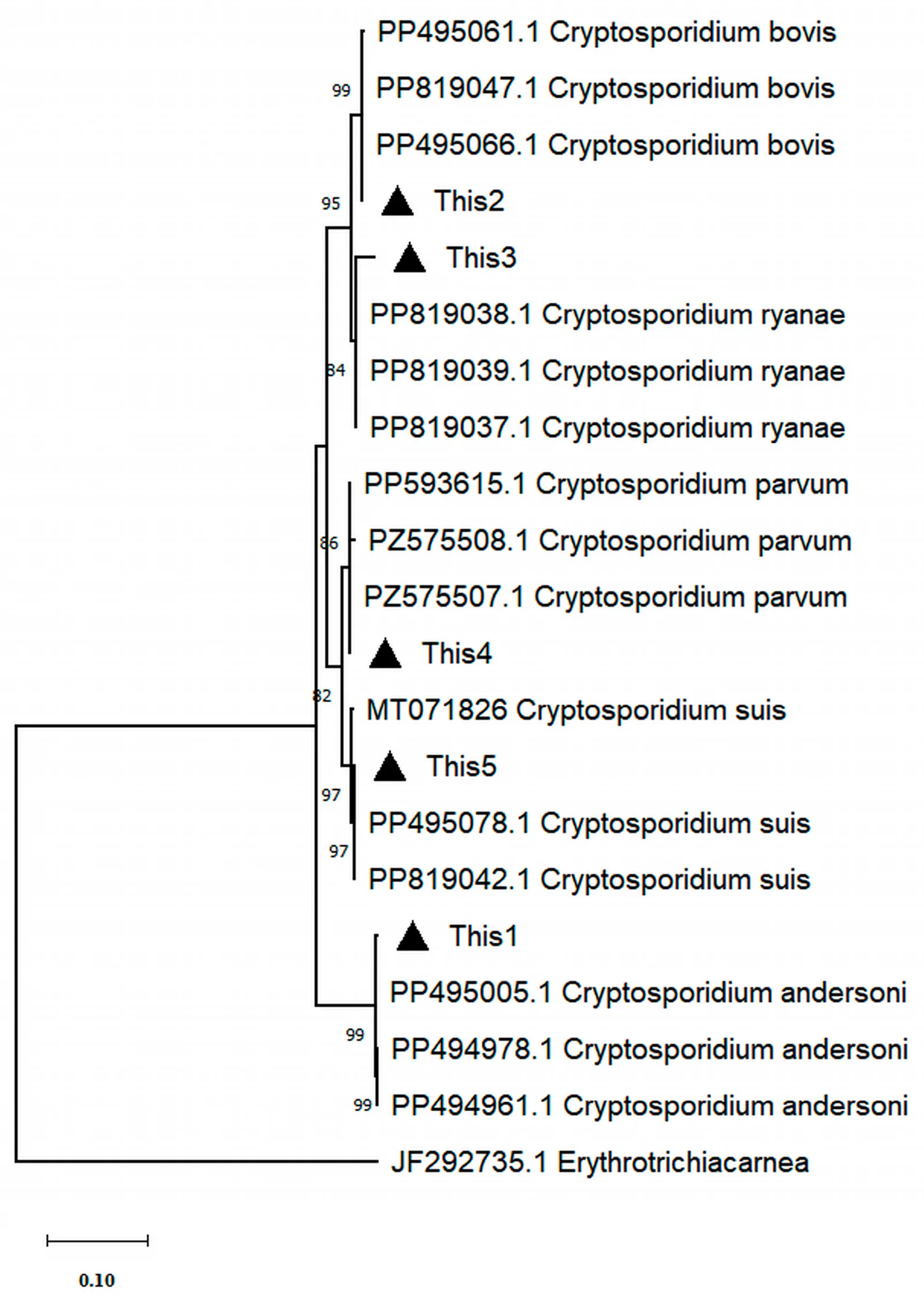
Maximum-likelihood phylogenetic tree of *Cryptosporidium* species based on SSU rRNA gene sequences. Sequences identified in this study are indicated by black triangles (▲). Bootstrap values ≥ 75% are presented at the nodes. The evolutionary history was inferred using the maximum likelihood method based on the Tamura three-parameter substitution model.

Notably, *C. suis* was detected in three samples (1.52% of positive cases and 0.59% of total samples). BLAST analysis of the SSU rRNA gene sequences revealed high degree of nucleotide identity to *C. suis* reference strains. Isolate Z-196 (GenBank PZ448284.1, 557 bp) exhibited 99.45% nucleotide identity with *C. suis* isolate NMG-168 (PP495078.1, 580 bp). Isolate Z-197 (PZ448285.1, 558 bp) exhibited 100% identity with isolate NMG-168 (PP495078.1, 580 bp) and 99.64% identity with *C. suis* voucher HS41 (PP819042.1, 562 bp). Isolate Z-198 (PZ448286.1, 558 bp) demonstrated 99.27% identity with isolate NMG-168 (PP495078.1, 580 bp). To confirm the accuracy of species identification by BLAST and visualize the evolutionary associations among the detected isolates and reference strains, phylogenetic analysis was conducted using SSU rRNA gene sequences. The maximum-likelihood phylogenetic tree (Figure 1) demonstrated that all five species identified in this study (*C. andersoni*, *C. parvum*, *C. bovis*, *C. ryanae*, and *C. suis*) formed distinct monophyletic clades with their corresponding GenBank reference sequences. All sequences obtained in this study (denoted by black triangles) tightly clustered with the reference strains of the same species, with bootstrap support values of ≥75% for all major nodes, thereby confirming the reliability of the BLAST-based species assignment. The three *C. suis* sequences identified in this study clustered with the *C. suis* reference strain (NMG-168, PP495078.1) with 97% bootstrap support, forming a clade that was clearly separated from *C. parvum*, *C. andersoni*, and other bovine *Cryptosporidium* species. This phylogenetic evidence further supports the identification of *C. suis* in dairy cattle in the present study.

In spring, the positive samples were distributed as follows: *C. andersoni* (*n* = 26), *C. parvum* (*n* = 12), *C. bovis* (*n* = 6), *C. ryanae* (*n* = 4), and *C. suis* (*n* = 1). In summer, the following distribution of positive samples was observed: *C. andersoni* (*n* = 2), *C. parvum* (*n* = 1), *C. bovis* (*n* = 7), and *C. ryanae* (*n* = 8). In autumn, the distribution of positive samples was as follows: *C. andersoni* (*n* = 34), *C. bovis* (*n* = 15), and *C. ryanae* (*n* = 10). In winter, the positive samples were distributed as follows: *C. andersoni* (*n* = 30), *C. parvum* (*n* = 11), *C. bovis* (*n* = 19), *C. ryanae* (*n* = 10), and *C. suis* (*n* = 2) (Table 2). Significant seasonal differences were observed for *C. andersoni* (χ^2^ = 27.759, df = 3, *p* < 0.001), *C. bovis* (χ^2^ = 9.994, df = 3, *p* = 0.019), and *C. parvum* (χ^2^ = 19.304, df = 3, *p* < 0.001): the prevalence of *C. andersoni* was lowest in summer (1.80%) and peaked in autumn (26.15%), whereas *C. parvum* was detected mainly in spring (8.96%) and winter (8.46%) and was absent in autumn. No significant seasonal variation was found for *C. ryanae* (*p* = 0.323).

In preweaned calves, the positive samples were distributed as follows: *C. andersoni* (*n* = 14), *C. parvum* (*n* = 9), *C. bovis* (*n* = 8), and *C. ryanae* (*n* = 8). In postweaned calves, the distribution of positive samples was as follows: *C. andersoni* (*n* = 24), *C. parvum* (*n* = 4), *C. bovis* (*n* = 16), and *C. ryanae* (*n* = 10). In young cattle, the following distribution was found: *C. andersoni* (*n* = 27), *C. parvum* (*n* = 7), *C. bovis* (*n* = 13), *C. ryanae* (*n* = 10), and *C. suis* (*n* = 2), and in adult cattle, the distribution was as follows: *C. andersoni* (*n* = 27), *C. parvum* (*n* = 4), *C. bovis* (*n* = 10), *C. ryanae* (*n* = 4), and *C. suis* (*n* = 1) (Table 2). Among the age groups, only *C. parvum* showed a significant difference in prevalence (χ^2^ = 8.757, df = 3, *p* = 0.030), being highest in preweaned calves (10.59%); *C. andersoni* (*p* = 0.934), *C. bovis* (*p* = 0.580), and *C. ryanae* (*p* = 0.174) were evenly distributed across all age groups. *C. suis* (*n* = 3) was detected only in young cattle (*n* = 2) and adult cattle (*n* = 1) in spring (*n* = 1) and winter (*n* = 2) and was not subjected to statistical testing. 

Among diarrheic cattle, the positive samples were distributed as follows: *C. andersoni* (*n* = 28), *C. parvum* (*n* = 10), *C. bovis* (*n* = 16), *C. ryanae* (*n* = 13), and *C. suis* (*n* = 1). In nondiarrheic cattle, the positive samples comprised *C. andersoni* (*n* = 64), *C. parvum* (*n* = 14), *C. bovis* (*n* = 31), *C. ryanae* (*n* = 19), and *C. suis* (*n* = 2) (Table 3). No statistically significant differences were observed between the two groups for any species (*p* > 0.05): *C. andersoni* (16.67% vs. 18.99%, *p* = 0.544), *C. bovis* (9.52% vs. 9.20%, *p* = 1.000), *C. ryanae* (7.74% vs. 5.64%, *p* = 0.438), *C. parvum* (5.95% vs. 4.15%, *p* = 0.381), and *C. suis* (0.60% vs. 0.59%, *p* = 1.000).

Among the 24 *C. parvum*-positive samples, gp60 sequencing revealed two distinct subtypes: IIdA20G1 (79.17%, 19/24) and IIdA19G1 (20.83%, 5/24). By season, IIdA20G1 was detected in spring (*n* = 8) and winter (*n* = 11), whereas IIdA19G1 was detected in spring (*n* = 4), summer (*n* = 1); no *C. parvum* was detected in autumn. By age group, IIdA20G1 was the predominant subtype and was detected in preweaned calves (*n* = 8), postweaned calves (*n* = 3), young cattle (*n* = 5), and adult cattle (*n* = 3). IIdA19G1 was detected in preweaned calves (*n* = 1), postweaned calves (*n* = 1), young cattle (*n* = 2), and adult cattle (*n* = 1). Among diarrheic cattle, IIdA20G1 was detected in 10 samples and IIdA19G1 in 3 samples. Among nondiarrheic cattle, IIdA20G1 was detected in 9 samples and IIdA19G1 in 2 samples (Table 4).

## 4. Discussion

### 4.1. Prevalence of Cryptosporidium spp.

The 39.21% infection rate in this single intensive dairy herd was higher than the global pooled prevalence of 26.5% [11]. Compared with other countries, our figure was also higher than those reported in Egypt (13.61%) [24], the United States (11.90%) [25], the Czech Republic (21.50%) [26], Argentina (25.5%) [27], South Korea (13.90%) [28], Canada (27.40%) [29], and Denmark (23.24%) [30], but lower than the rates from Spain (49.20%) and Austria (55.40%) [31,32]. In China, this prevalence was higher than the national cattle herd baseline (17.0%) and also higher than provincial rates from Shanxi (11.19%) [33], Jiangxi (12.80%) [34], Guangdong (4.83%) [35], Heilongjiang (5.50%) [36], Yunnan (15.80%) [37], Anhui (2.40%) [38], Shaanxi (3.40%) [39], Qinghai (28.50%) [40], Sichuan (14.40%) [41], Xinjiang (3.80%) [42], Gansu (4.20%) [43], and Ningxia (19.38%) [44]. It was also higher than the 29.90% reported in earlier Inner Mongolia surveys [45], and only lower than the 48.7% from Xinjiang [18]. This wide variation reflects the heterogeneity in *Cryptosporidium* burden across different geographic and management settings.

Notably, this study was conducted on a single intensive dairy farm in Hohhot, Inner Mongolia. Herd size, management practices, housing conditions, and biosecurity protocols at this farm may differ from those at other farms within Inner Mongolia or northern China. Consequently, the prevalence, species distribution, and seasonal patterns observed in this study may not be representative of the broader regional dairy cattle population. Therefore, caution should be exercised when extrapolating these findings to other farms, geographic regions, or production systems (e.g., pasture-based or smallholder farms). Multifarm studies covering diverse management systems are warranted to confirm the regional epidemiological patterns of *Cryptosporidium* infection in Inner Mongolia.

### 4.2. Seasonal Prevalence of Cryptosporidium spp.

The prevalence of *Cryptosporidium* spp. was the lowest in summer (16.22%), gradually increased through autumn (45.38%) and winter (55.38%), and then decreased again in spring (36.57%). Iran data reported prevalence rates of 14%, 11%, 18%, and 21% across spring, summer, autumn, and winter, respectively [46]. Egyptian surveys also recorded a higher prevalence in autumn (51.28%) and winter (40.09%) than in spring (37.73%) and summer (38.01%) [47]. Xinjiang reports, by contrast, showed higher prevalence in spring (50.0%) and summer (56.8%) than in autumn (41.7%) and winter (46.8%) [18].

Because the survey was confined to one farm, the seasonal pattern may reflect management as much as climate. Summer lows align with oocyst sensitivity to heat, desiccation, and UV; winter highs coincide with indoor confinement, higher stocking density, poorer ventilation, and frozen ground that traps manure. Autumn–winter calving on this farm also concentrated susceptible neonates during the high-risk period. Cross-study differences in housing, sampling, and herd structure may likewise shift seasonal curves.

### 4.3. Prevalence of Cryptosporidium spp. Among Different Age Groups and Symptoms

Analysis by age group showed the highest infection rates in preweaned calves (45.88%) and young cattle (43.07%), with lower rates in postweaned calves (39.42%) and adult cattle (31.51%). This pattern is similar to the age-related distribution described in a review of Chinese cattle herds, which reported peak prevalence in preweaned calves (19.5%), followed by young cattle (10.69%), postweaned animals (9.0%), and adults (4.94%) [7]. These results are also consistent with earlier reports from Inner Mongolia, where the prevalence was 27.18% in preweaned calves, 43.81% in postweaned calves, 37.10% in young cattle, and 18.50% in adults [45]. However, Guangdong Province showed a different pattern, with the lowest prevalence in young cattle (1.48%) and adults (2.06%), whereas preweaned (6.4%) and postweaned (6.19%) calves showed comparable rates [35].

Diarrheic and nondiarrheic cattle showed comparable infection rates of 40.48% and 38.58%, and there was no significant difference between them. This is consistent with most published reports, which generally note marginally higher rates in diarrheic animals. But marked disparities have been documented in Colombia (52.2% versus 19.9%) [48] and Cameroon (74.72% versus 35.87%) [49]. Earlier studies in Inner Mongolia also reported higher rates in diarrheic cattle (37.67% versus 26.74%) [45]. However, Ghanaian surveys showed the opposite pattern, with nondiarrheic cattle having a higher prevalence (26.1%) than diarrheic cattle (12.2%) [50].

The present study investigated the associations between *Cryptosporidium* infection and season, age group, and diarrhea status via univariable analyses (chi-square test and pairwise odds ratios). Season was significantly associated with *Cryptosporidium* prevalence (χ^2^ = 41.37, df = 3, *p* < 0.001), whereas age group (χ^2^ = 6.079, df = 3, *p* = 0.108) and diarrhea status (χ^2^ = 0.100, df = 1, *p* = 0.752) were not. However, these analyses did not account for potential confounding or interactions among season, age, and diarrhea status. Multivariable logistic regression with interaction terms (e.g., season × age, season × diarrhea status) was not conducted. Consequently, the independent effects of each factor and their potential interactions remain unclear.

### 4.4. Prevalence of Cryptosporidium spp. and Subtypes

In addition to the four common *Cryptosporidium* spp. in cattle reported globally and in China, viz., *C. andersoni*, *C. bovis*, *C. ryanae*, and *C. parvum*, the present study identified *C. suis* for the first time in China. Reports from Poland also indicate that *C. suis* has been detected in cattle [51]. Owing to the low prevalence (0.59%) and small sample number (*n* = 3), several alternative explanations for the presence of *C. suis* in this dairy herd must be considered. First, although the study farm did not house pigs or report direct contact with pig operations, the possibility of indirect transmission through contaminated water sources cannot be completely ruled out. *C. suis* oocysts are environmentally robust and may persist in surface water, groundwater, or irrigation sources if upstream or adjacent pig farms discharge manure into shared watersheds. In this study, the water supply of the farm—whether from municipal sources, wells, or surface water—was not tested for *Cryptosporidium* oocysts, and therefore this potential route of introduction remains unverified but plausible.

From a seasonal perspective, *C. andersoni* infections are predominant in spring, autumn, and winter, whereas *C. bovis* and *C. ryanae* infections are more common in summer. Xinjiang data differ, with *C. parvum* identified as the dominant species [18]. In studies from Henan Province, *C. parvum* was found to be dominant in autumn, *C. bovis* was identified as dominant in both autumn and winter, and both species were prevalent in spring [52].

From the perspective of age, *C. andersoni* was identified as the dominant species among all four age groups, a finding that is consistent with a previous study reporting that *C. andersoni* was dominant in calves before and after weaning and in adult cattle [7]. Similarly, another study from Yunnan Province indicated that *C. andersoni* was the dominant species in cattle across all age groups [8]. In this study, *C. andersoni* was the dominant species across all age groups (46.47% of positive samples), which contrasts with the age-associated distribution pattern documented in many global studies, where *C. parvum* generally dominates in preweaned calves, *C. bovis* and *C. ryanae* in postweaned calves, and *C. andersoni* in older cattle. First, the intensive management system in this farm, including shared water troughs, and communal housing for different age groups, may facilitate cross-age transmission of *C. andersoni*, which is considered a cattle-adapted species with low host specificity within bovines. Second, the adult cattle population on this farm may have served as a persistent reservoir for *C. andersoni*, continuously exposing younger animals via environmental contamination. Third, the higher infection pressure from *C. andersoni* in the adult herd may have suppressed or concealed the typical neonatal dominance of *C. parvum.*

*C. andersoni* was dominant in both diarrheic and nondiarrheic cattle, matching observations from Shanxi [33]. Cyprus data identified *C. parvum* as the dominant species in both groups [53]. Earlier Inner Mongolian surveys reported different species: central herds showed *C. bovis* in diarrheic cattle but *C. andersoni* in nondiarrheic cattle [45], and western herds showed *C. parvum* and *C. bovis* as the dominant species in diarrheic and nondiarrheic cattle, respectively [54].

*C. parvum* is the major pathogenic and zoonotic species in dairy cattle, and threatens the dairy industry and public health security. Moreover, >90% of *Cryptosporidium* infections in humans are caused by *C. parvum* and *C. hominis*. In the present study, two subtypes were detected in our isolates: IIdA20G1 (19/24) and IIdA19G1 (5/24). Cattle *C. parvum* worldwide falls into three gp60 families—IIa, IId, and IIl. IIa dominates every continent except Africa and Asia. IId has been found in every surveyed region except the United States, and China holds the largest share. IIl remains restricted to Europe [11]. Among the IId family, the four most prevalent genotypes are IIdA19G1, IIdA20G1, IIdA15G1, and IIdA14G. To date, eight *C. parvum* subtypes (IIdA14G1, IIdA15G1, IIdA17G1, IIdA18G1, IIdA19G1, IIdA20G1, IIdA21G1, and IIdA24G2) have been identified in dairy cattle in China [13,55,56]. IIdA19G1 is the most prevalent sublineage in China [57,58], as confirmed by studies conducted in Henan [52], Shanghai [59], Heilongjiang, and Guangdong [60,61]. Furthermore, a study from Inner Mongolia indicated that all cases of *C. parvum* were identified as being infected with the IIdA19G1 subtype [45]. Infections with the *C. parvum* subtype IIdA20G1 have also been reported in Hebei [62] and Heilongjiang [55]. These data indicate that the distribution of *C. parvum* subtypes varies across different regions, with IIdA19G1 being the most prevalent subtype in China.

Both IIdA19G1 and IIdA20G1 detected in this study have been reported in human infections, supporting their zoonotic potential. IIdA19G1 has been identified in hospitalized children in China and in patients with HIV/AIDS in Ethiopia and Iran [63,64,65]. IIdA20G1 has been reported in human infections in Iran, including patients with gastrointestinal illnesses and HIV-infected individuals [65,66]. These findings highlight the zoonotic potential of the *C. parvum* subtypes circulating in the studied dairy herd and highlight the need for enhanced surveillance at the human–animal interface.

These zoonotic subtypes raise the possibility of human exposure, yet without concurrent human or environmental samples we cannot trace transmission routes. Their presence on the farm is therefore a warning sign rather than proof of spillover. One Health follow-up combining human and environmental sampling would be needed to quantify real risk.

## 5. Conclusions

To the best of our knowledge, this is the first systematic investigation on the seasonal distribution of *Cryptosporidium* spp. in cattle of Inner Mongolia. The results demonstrate that *Cryptosporidium* is prevalent in cattle across Inner Mongolia throughout the year, with the lowest prevalence observed in summer, gradually increasing in autumn and winter, and reaching the highest level in winter. *C. andersoni* remains the dominant cattle-infecting species in Inner Mongolia, and *C. suis* was detected in Chinese cattle herds for the first time in this study. Both of the two *C. parvum* subtypes detected in this study were zoonotic genotypes. Currently, there is insufficient systematic research on the seasonal distribution characteristics of *Cryptosporidium* in cattle, and further studies are required to determine the associated zoonotic risks and the impact of these pathogens on public health. Owing to the single-farm design, further multifarm studies are warranted to determine whether these patterns are representative of the broader regional dairy industry.

## Figures and Tables

**Table 1:** Prevalence of *Cryptosporidium* spp. infection in dairy cattle by season, age group, and clinical manifestation.

Variable	Category	No. Examined	No. Positive	Prevalence (%)	χ^2^ (df)	*p*-Value	OR (95% CI) ^a^
Season	Spring	134	49	36.57	41.37 (3)	<0.001	2.98 (1.61–5.51)
	Summer (Ref.)	111	18	16.22			1.00
	Autumn	130	59	45.38			4.29 (2.33–7.91)
	Winter	130	72	55.38			6.41 (3.48–11.83)
Age group	Preweaned calves (0–60 days)	85	39	45.88	6.079 (3)	0.108	1.84 (1.06–3.20)
	Postweaned calves (61–180 days)	137	54	39.42			1.41 (0.87–2.31)
	Young cattle (181–360 days)	137	59	43.07			1.64 (1.01–2.67)
	Adult cattle (≥361 days) (Ref.)	146	46	31.51			1.00
Clinical manifestation	Diarrheic	168	68	40.48	0.100 (1)	0.752	1.08 (0.74–1.58)
	Nondiarrheic (Ref.)	337	130	38.58			1.00
Total	—	505	198	39.21	—	—	—

^a^ OR (95% CI): odds ratio with 95% confidence interval calculated against the reference (Ref.) category of each variable; 1.00 denotes the reference category. χ^2^ (df): chi-square statistic with degrees of freedom; *p*-values are from chi-square tests for overall comparisons within each variable.

**Table 2:** Prevalence of *Cryptosporidium* species in dairy cattle by season and age group.

***Cryptosporidium* Species** ^a^	**Season, *n* (%)**				**χ^2^ (df) ^b^**	***p*** **-Value ^b^**	**Age Group, *n* (%) ^d^**				**χ^2^ (df) ^b^**	***p*** **-Value ^b^**
	**Spring (*n* = 134)**	**Summer (*n* = 111)**	**Autumn (*n* = 130)**	**Winter (*n* = 130)**			**Preweaned (*n* = 85)**	**Postweaned (*n* = 137)**	**Young (*n* = 137)**	**Adult (*n* = 146)**		
*C. andersoni*	26 (19.40)	2 (1.80)	34 (26.15)	30 (23.08)	27.759 (3)	<0.001	14 (16.47)	24 (17.52)	27 (19.71)	27 (18.49)	0.431 (3)	0.934
*C. bovis*	6 (4.48)	7 (6.31)	15 (11.54)	19 (14.62)	9.994 (3)	0.019	8 (9.41)	16 (11.68)	13 (9.49)	10 (6.85)	1.964 (3)	0.580
*C. ryanae*	4 (2.99)	8 (7.21)	10 (7.69)	10 (7.69)	3.483 (3)	0.323	8 (9.41)	10 (7.30)	10 (7.30)	4 (2.74)	4.965 (3)	0.174
*C. parvum*	12 (8.96)	1 (0.90)	0 (0)	11 (8.46)	19.304 (3)	<0.001	9 (10.59)	4 (2.92)	7 (5.11)	4 (2.74)	8.757 (3)	0.030 ^c^
*C. suis*	1 (0.75)	0 (0)	0 (0)	2 (1.54)	NT	NT	0 (0)	0 (0)	2 (1.46)	1 (0.68)	NT	NT
**Total**	49 (36.57)	18 (16.22)	59 (45.38)	72 (55.38)	41.37 (3)	<0.001	39 (45.88)	54 (39.42)	59 (43.07)	46 (31.51)	6.079 (3)	0.108

^a^ *Cryptosporidium* species are listed in descending order of overall prevalence. Prevalence (%) = number of samples positive for each species/total number of samples examined in that group. ^b^ Chi-square test for differences in prevalence among the four seasons or the four age groups. NT: not tested owing to the small number of *C. suis*-positive samples (*n* = 3); data are presented descriptively. ^c^ One expected cell count was <5; the result was confirmed by Monte Carlo simulation (20,000 permutations) of Fisher’s exact test (*p* = 0.030). ^d^ Age groups: preweaned calves (0–60 days), postweaned calves (61–180 days), young cattle (181–360 days), and adult cattle (≥361 days).

**Table 3:** Prevalence of *Cryptosporidium* species in diarrheic and nondiarrheic dairy cattle.

*Cryptosporidium* Species	Diarrheic Cattle (*n* = 168), *n* (%)	Nondiarrheic Cattle (*n* = 337), *n* (%)	OR (95% CI) ^a^	*p*-Value ^b^
*C. andersoni*	28 (16.67)	64 (18.99)	0.85 (0.52–1.39)	0.544
*C. bovis*	16 (9.52)	31 (9.20)	1.04 (0.55–1.96)	1.000
*C. ryanae*	13 (7.74)	19 (5.64)	1.40 (0.68–2.92)	0.438
*C. parvum*	10 (5.95)	14 (4.15)	1.46 (0.63–3.36)	0.381
*C. suis*	1 (0.60)	2 (0.59)	1.00 (0.09–11.14)	1.000
Total	68 (40.48)	130 (38.58)	1.08 (0.74–1.58)	0.700 ^c^

^a^ OR (95% CI): odds ratio with 95% confidence interval for diarrheic versus nondiarrheic cattle. ^b^ Two-sided Fisher’s exact test comparing the prevalence of each *Cryptosporidium* species between diarrheic and nondiarrheic cattle. ^c^ The overall comparison was also non-significant by the chi-square test (χ^2^ = 0.100, df = 1, *p* = 0.752; see Table 1). Prevalence (%) = number of positive samples/total number of samples examined in each group.

**Table 4:** Distribution of *Cryptosporidium parvum* gp60 subtypes by season, age group, and clinical manifestation.

Variable	Category	IIdA19G1 (*n*)	IIdA20G1 (*n*)	Total (*n*)
Season	Spring	4	8	12
	Summer	1	0	1
	Autumn	0	0	0
	Winter	0	11	11
Age group	Preweaned calves (0–60 days)	1	8	9
	Postweaned calves (61–180 days)	1	3	4
	Young cattle (181–360 days)	2	5	7
	Adult cattle (≥361 days)	1	3	4
Clinical manifestation	Diarrheic	3	10	13
	Nondiarrheic	2	9	11
Total	—	5	19	24

Formal statistical testing was not performed owing to the small number of *C. parvum*-positive samples (*n* = 24); counts are presented descriptively.

## Data Availability

The nucleotide sequences obtained in this study have been deposited in the GenBank database under the accession numbers PZ448089–PZ448286 and PZ669771-PZ669794.

## Abbreviations

SSU rRNA	small subunit ribosomal RNA
CI	confidence interval
OR	odds ratio
gp60	60kDaglycoprotein
PCR	Polymerase Chain Reaction
